# A Case of Obstructive Sleep Apnea and Assessments of Fitness for Work

**DOI:** 10.1186/2052-4374-26-7

**Published:** 2014-04-25

**Authors:** Sukwoo Hong, Yewon Kim, Ji-Young Ryu, Sangyoon Lee, Byung-Chul Son, Chae-Kwan Lee, Dae-Hwan Kim

**Affiliations:** 1 Department of Occupational and Environmental Medicine, Inje University Haeundae Paik Hospital, 875 Heaundae-ro, Haeundae-Gu, Busan 612-862, South Korea; 2 Department of Occupational and Environmental Medicine, College of Medicine & Institute of Environmental and Occupational Medicine, Inje University, 75 Bokji-ro, Busanjin-gu, Busan, South Korea

**Keywords:** Obstructive sleep apnea, Fitness for work, Polysomnography

## Abstract

**Background:**

Obstructive sleep apnea is a common sleep disorder that can cause excessive daytime sleepiness and impairment of cognition. These symptoms may lead to the occurrence of occupational accidents in workers with obstructive sleep apnea.

**Case presentation:**

A 36-year-old man who worked as a dimensional control surveyor caused a vehicle accident while he was driving at the work site. Although he experienced loss of consciousness at the time of the accident, he had no other symptoms. His brain computed tomography and laboratory test did not show any specific findings. Medical tests were conducted to evaluate his fitness for work. Decreased sleep latency was observed on the electroencephalography image, which is suggestive of a sleep disorder. He frequently experienced daytime sleepiness and his Epworth sleepiness score was 13. The polysomnography showed a markedly increased apnea-hypopnea index of 84.3, which led to a diagnosis of severe obstructive sleep apnea. The patient was advised to return to work only when his obstructive sleep apnea improved through proper treatment.

**Conclusion:**

Proper screening for obstructive sleep apnea among workers is important for preventing workplace accidents caused by this disorder, but screening guidelines have not yet been established in Korea. An effort toward preparing practical guidelines for obstructive sleep apnea is needed.

## Background

Obstructive sleep apnea (OSA) is a common sleep disorder that occurs owing to repetitive partial or complete upper airway obstruction, thus blocking the airflow and disturbing the gas exchange ratio (respiratory quotient). This airflow disruption reduces blood oxygen saturation and induces changes in the cardiovascular system. OSA also can result in repetitive arousal, sleep fragmentation, snoring, and excessive daytime sleepiness.

In the U.S., the prevalence of OSA accompanied by complaints of sleepiness was reported as 4.0% and 2.0% in adult men and women, respectively [1]. Another study conducted in the United States reported that the prevalence in adult men and women was 3.9% and 1.2%, respectively [2]. A study conducted in an Australian population reported a prevalence of 3.1% for OSA in adult men [3]. The prevalence of OSA in adult men versus women is 7.5% versus 4.5% in India [4], and is 4.1% versus 2.1% in Hong Kong [5,6]. A study conducted in South Korea [7] showed a prevalence of 4.5% and 3.2% among middle-aged adult men and women. These results indicate that the prevalence of this disease shows similar tendencies in different countries.

Studies on OSA in the occupational setting have been conducted in many countries. According to a study conducted in 2002 involving 1,391 commercial vehicle drivers in Pennsylvania, U.S.A., 17.6%, 5.8%, and 4.78% of drivers showed mild, moderate, and severe sleep apnea, respectively [8]. In Hong Kong, 51 commercial bus drivers were randomly recruited for a test with a portable sleep monitoring device (PMD), and 10 were diagnosed with sleep apnea [9]. Similar findings were observed in truck drivers in Guangzhou, China, in which 125 (37.3%) out of 335 drivers were shown to have OSA [10]. In Italy in 2004, out of 120 men of working age who responded to a survey on daytime sleepiness, 20 scored over 14 points on the Epworth Sleepiness Scale (ESS) and underwent polysomnography (PSG), which revealed that all of them had moderate or severe OSA [11]. A study with 21 shiftwork nurses in the U.S.A. showed that 9 of the subjects had sleep-disordered breathing, whereas 3 of them had sleep apnea [12].

Symptoms of excessive sleepiness and reduced cognitive function, which OSA patients are likely to develop, can lead to decreased mental processing, memory, and concentration, as well as an increase in reaction time, to the extent of impairing work productivity [13,14]. These symptoms can prevent these subjects from responding appropriately to situations requiring immediate judgment and reaction, which in turn may result in accidents [15,16]. According to the 2012 European Statistics on Occupational Accidents, 29% of fatal accidents and 4% of non-fatal accidents were caused by loss of vehicle control [17]. Accidents occurring on industrial sites can cause death and injury to those responsible for the accidents as well as to the people around them. Further, accidents caused by those working in safety-sensitive occupations, such as commercial vehicle drivers transporting passengers or large-scale freights, heavy-equipment operators at construction sites and other industrial sites, railway workers, people working at a height, and workers in airline or shipping companies, can result in a heavy toll on human lives and assets.

Despite these potential risks caused because of OSA in industrial sites and in public health, no study to date has been performed with respect to the evaluation, management, treatment, and assessment of fitness for the OSA-related performance of the working population in Korea. In response to the need for increasing the general interest in OSA-related occupational and environmental medicine, we report a case of OSA in a worker who caused a vehicle accident at the workplace.

## Case presentation

The patient was a 36-year-old Southeast Asian man who was an employee of a multinational corporation. At the time of the accident, he had been working as a dimensional control surveyor for module production at the construction of an offshore plant for 7 months at a Korean shipyard.

He caused an accident on the premises of the shipyard on December 4, 2012, at around 2 p.m., by crashing into a concrete wall after colliding with a motorcycle parked on the street. He presented to the emergency department of a nearby university hospital with the help of the company health officer. No conspicuous physical symptoms were observed except for mild tenderness on the right upper arm, but he had no memory of the period immediately before and after the accident. He had been wearing his safety belt, and, according to the statement of a worker who witnessed the accident, he was driving at a speed of ≤ 30 km/h, which is the speed limit on the premises. The accident itself was negligible, but he was unconscious at the time of the accident and he recovered consciousness only after a worker at the site knocked on the window approximately 5 min after the accident.

The results of a blood test, radiography of the right upper arm, and brain computed tomography (CT) did not reveal any abnormalities. The patient refused to undergo a more detailed examination to determine the cause of his loss of consciousness (LOC), and he was discharged without further treatment. The patient revisited the university hospital 3 days later, and no abnormalities were detected with the neurological examination conducted at the outpatient care of the neurosurgery department. Although the patient did not have any LOC-associated medical history, the medical team of the company decided to limit his work involving safety-sensitive activities such as driving and working at a height, in confined spaces, and with electricity, because of the risk of recurrence. He was referred to the Department of Occupational and Environmental Medicine of our hospital on December 31, 2012, for an assessment of fitness for work.

The following facts were communicated in the interview held on the day of his presentation. The patient had been working at a Korean shipyard as an employee of a multinational corporation since May 2012. As a dimensional control surveyor, he was responsible for module production, process management, and quality control in a liquefied natural gas plant. He started work between 7 and 8 a.m., and worked 9 hours a day, excluding a lunch break, and spent about 40% of his working hours at the site. His on-site activities consisted mainly of visiting the module production sites dispersed across various workplaces within the shipyard and collecting the production process data by using a laser scanner. Upon returning to the office, he would reconstruct the 10–15 datasets collected during his visits by using an elaborate 3-dimensional data computer program, and he used the 3-dimensional data to check whether the major module structures or complicated piping arrangements were produced within the error ranges admissible by the design. He then corrected the design drawing based on the detected production errors deviating from the permitted ranges, and had the work verified by the dimensional control manager. He spent 40–45% of his total working hours in the office, and all module production sites were within a distance of several kilometers. The shipyard premises contained traffic signs specifying a speed limit of 30 km/h. Because of the traffic caused by the transport of material and heavy equipment, it took approximately 5–10 min to move from one module to the next, and he spent about 15% of his working hours moving around by car. He alternated between 8 working weeks without a break and 2 off-work weeks in his home country, and he did not engage in overtime or shiftwork.

His baseline physical characteristics were as follows: height, 180 cm; weight, 112 kg; body mass index (BMI), 34.6 kg/m^2^; blood pressure, 145/95 mmHg; body temperature, 36.5°C; pulse, 63 beats/min; and respiratory rate, 22 breaths/min. He had been consuming medication for hypertension for a year without any other notable medical history; he did not smoke or drink.

A blood test, peripheral blood smear examination (PBS), urine test, electrocardiography, and treadmill test were conducted to detect any possible medical condition that may have been the cause of LOC. The blood test revealed a fasting blood glucose level of 108 mg/dL; the test values for glycated hemoglobin and total red blood cell count as well as the levels of hemoglobin, hematocrit, blood electrolytes, blood urea nitrogen, and creatinine were all normal. The results of the PBS and urine examinations as well as electrocardiography did not reveal any abnormalities. No abnormalities were observed during the exercise portion of the treadmill test; however, the test was discontinued 2 min and 10 s after it began, when his systolic blood pressure exceeded 200 mmHg. Electroencephalography (EEG) and temporal lobe epilepsy magnetic resonance imaging (TLE-MRI) were performed to detect any possible neurological cause of LOC. The EEG and TLE-MRI readings did not reveal any abnormalities that were likely to cause epilepsy or syncope, but we decided to perform a more detailed examination for sleep disorders when we observed short sleep latency on the EEG result.

When the patient presented again to our hospital on January 14, 2013, we performed another interview that was focused on sleep problems in addition to detailed anthropometrics and ESS. His neck circumference (NC) measured 46 cm (18.1 inch) and ESS was 13 points (Table 1). He usually went to bed between 11 and 12 p.m. and woke up at around 6 a.m. In his home country, his wife had complained about his snoring, and he used to hold his breath during sleep and subsequently needed to gasp for breath. As he reported that he took a nap for 20–30 min to prevent sleepiness in the afternoon hours after lunch, PSG was performed to determine whether he had a sleep disorder.

**Table 1:** Epworth Sleepiness Scale score of the patient

**Likelihood of falling asleep in the following situations**	
Chance of dozing (0–3)	
0 = would never doze off	
1 = slight chance of dozing	
2 = moderate chance of dozing	
3 = high chance of dozing	
**Score for each situation**	
Sitting and reading	1
Watching TV	1
Sitting, inactive, in a public place (e.g., a theater or a meeting)	2
As a passenger in a car for an hour without a break	2
Lying down to rest in the afternoon when circumstances permit	3
Sitting and talking to someone	0
Sitting quietly after a lunch without alcohol	2
In a car that is stopped for a few minutes in traffic	2
**Total**	13*

*A total score of 0–9 is normal, 10–15 is considered sleepy, and ≥ 16 is considered very sleepy.

**Figure 1:**
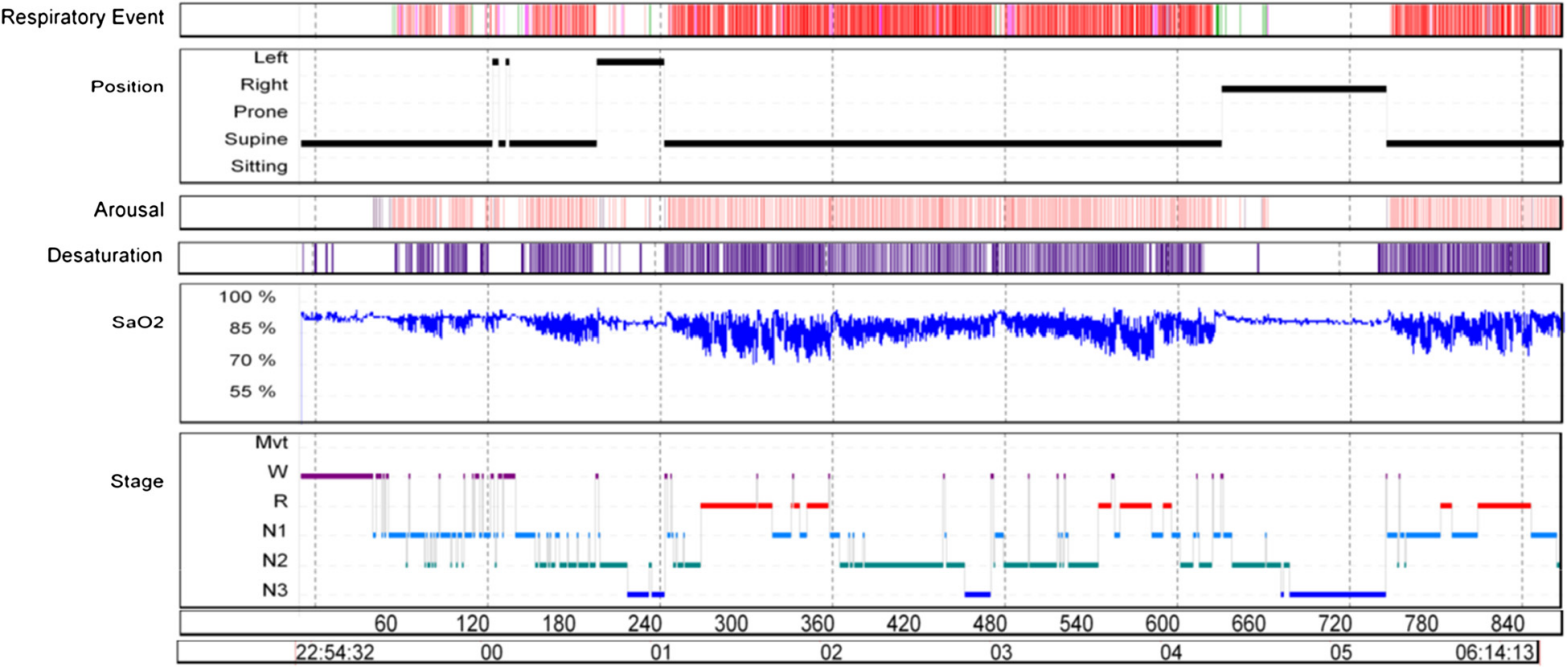
**An overnight summary view of polysomnography of the case shows respiratory events, arousals, desaturation events, arterial oxygen saturation, and sleep stages.** The sleep study revealed an AHI score of 84.3 and lowest oxygen saturation of 70%, which is consistent with a diagnosis of severe obstructive sleep apnea with desaturation. SaO_2_ = arterial oxygen saturation, AHI = apnea hypopnea index, Mvt = movement, W = waking state R = rapid eye movement sleep, and N1, N2, and N3 = non-REM sleep stages.

The PSG performed in our hospital on January 30, 2013, yielded the following results: sleep latency, 25 min; sleep efficiency, 88.6%; total sleep time, 388.5 minutes out of a total recording time of 438.5 minutes (Figure 1, Table 2). The sleep latency of rapid eye movement (REM) was 114 minutes, and the sleep architecture showed a substantially increase in stage N1, decrease in stage N2, and decrease in stage R. Frequent respiratory events and arousals were observed when he was sleeping in a supine position, but these were rare when he slept in a lateral decubitus position. Particularly, the respiratory events increased, and the recorded minimum blood oxygen saturation was 70% in REM sleep. The apnea-hypopnea index (AHI) was measured as 84.5, indicating severe sleep apnea.

**Table 2:** Summary of the respiratory events of the patient

	**Sleep stage**		**Position**		**Total**
	**NREM**	**REM**	**Non-supine**	**Supine**	
Sleep time (min.)	313	75.5	81.0	307.5	388.5
Apneas	391	120	7	504	511
Hypopneas	35	0	4	31	35
Apneas + Hypopneas	426	120	11	535	546
Apnea index	75.0	95.4	5.2	98.3	78.9
Hypopnea index	6.7	0	3.0	6.0	5.4
AHI score*	81.7	95.4	8.2	104.4	84.3

*AHI, Apnea hypopnea index; *REM*, Rapid eye movement; *NREM*, Non-rapid eye movement.

On the basis of the results of the interview and tests, we concluded that it was very likely that the accident was caused because of OSA while the patient was driving, and we recommended that the patient resume working only after the OSA was alleviated through continuous positive airway pressure (CPAP) or surgical intervention.

Relying on the results of the assessment of fitness for work, the company temporarily suspended the patient from work and decided to reinstate him to his usual duties when his OSA was alleviated. The treatment period for OSA was estimated to last for 2–3 months, and it was decided that the patient would receive the treatments in his home country, and his reinstatement to work would depend on the results of a reassessment of fitness for work.

## Conclusions

Apnea is defined as a state of a complete or a near-complete pause in breathing for over 10 s during sleep, irrespective of reduced blood oxygen saturation or sleep fragmentation [18]. The criteria for defining hypopnea as recommended by the American Academy of Sleep Medicine (AASM) is a ≥ 30% reduction in airflow for 10 s or longer associated with a ≥ 4% drop in oxygen saturation, but no consensus currently exists regarding its definition [19,20]. The apnea-hypopnea index (AHI) is the sum of apneas and hypopneas per hour during sleep. The diagnostic standard for OSA is an AHI ≥ 5 when accompanied by sleep-related symptoms or an AHI ≥ 15 when not accompanied by sleep-related symptoms. OSA severity is determined by considering the intensity of sleepiness and the AHI (5–14, mild; 15–30, moderate; and above 30; severe) [21,22].

OSA-related risk factors include obesity, male gender, and aging. The recent increase in obesity in the population, which is the major factor for sleep-disordered breathing, has prompted a rapid increase in the number of patients diagnosed with OSA. Approximately 70% of patients with OSA are estimated to be obese [23]. An increase or decrease in body weight is directly associated with an increase or decrease in the AHI score, and an increase of 10% body weight was reported to correspond to a 6-fold higher risk for developing moderate or severe OSA [24,25].

According to a previous study [26], NC is more strongly associated with OSA than is BMI. Specifically, an NC ≥ 17 inches for men and ≥ 16 inches for women were highly correlated with OSA [27]. However, a study conducted by Simpson et al. [28] reported that, while NC and the AHI score are closely related to OSA in women, abdominal circumference is more strongly associated with OSA than is NC in men.

Male sex is one of the risk factors for OSA, as confirmed by the results of a number of studies in which men showed a higher prevalence of OSA than women [29]. Further, the risk of OSA is higher in men than in women [30]. The sex-dependent variation may be explained by considering the anatomical differences between men and women, because men generally possess a longer airway and therefore have a higher tendency towards pharyngeal collapse [31]. Another study suggested that the sex-based discrepancy in OSA is attributed to the greater fat proportion around the upper airway in men compared to that in women [32].

Increasing age is related to a higher prevalence of OSA [33]. According to a study conducted by Ancoli-Israel et al. [34], the prevalence of OSA in the elderly population (≥ 65 years) was 3-fold higher than that in the middle-aged population, and the prevalence of sleep-disordered breathing among Japanese truck drivers was higher in the age bracket of 40–69 years than that in the 20–39 age bracket [35].

Although the patient in this reported case was not elderly (36 years), he had OSA risk factors such as obesity (BMI, 34.6 kg/m^2^) and a large NC (46 cm or 18.1 inches). His symptoms of snoring and daytime sleepiness could be attributed to OSA. Further, the patient’s underlying hypertension may have been induced or exacerbated because of the cardiovascular load related to sleep apnea because OSA is associated with cardiovascular disorders such as hypertension, arrhythmia, coronary artery disease, and abdominal aortic aneurysm [36,37]. As CPAP therapy is known to improve blood pressure [38], it was expected that CPAP would improve his hypertension and, subsequently, his OSA.

Individuals suspected to have OSA should first undergo a physical examination. BMI, blood pressure, and NC should be measured. Subsequently, the patient should be checked for anatomical abnormalities such as retrognathia, and a detailed examination of the upper airway, including the nasal cavity and oropharynx, should be performed for the OSA risk assessment.

Among the screening tests for OSA, questionnaire surveys can be conducted for individuals who are suspected of having OSA. The ESS is a widely used questionnaire survey method to measure subjective sleepiness (Table 1). The total score of the test ranges from 0 (minimum) to 24 (maximum). A total score of < 10 is considered normal and a score of ≥ 16 is indicative of excessive daytime sleepiness [39]. The Berlin questionnaire survey is also widely used to identify patients at risk for sleep apnea. In a study based on PSG, the sensitivity and specificity of ESS were estimated to be 0.66 and 0.48, respectively [40], and a study on the application of portable monitoring revealed sensitivity and specificity estimates of 0.77 and 0.89, respectively, for the Berlin questionnaire survey [41]. In a recent study, in which the responses of 12-h-shift nurses to the Berlin questionnaire were analyzed by using PSG, the sensitivity, specificity, and positive predictive value were estimated to be 0.33, 0.83, and 0.60, respectively, indicating that the sensitivity was lower compared to that of previous studies that were conducted in non-occupational subjects [12].

These questionnaire surveys are mainly used for clinical purposes and not in occupational settings. As the subjects of the studies conducted until now have been patients from sleep laboratories and the general population, research applicable to various occupational settings is needed. The work characteristics of the targeted occupations should be taken into consideration in order for such tools to be used for screening for sleep disorders among workers of various occupational groups, such as night-shift workers.

PSG is a standard test used for diagnosing and classifying the severity of sleep apnea. PSG records the subject’s sleep status, respiration rate, electrocardiogram results, electrical activity of the muscles, body position, oxygen level, and snoring occurrence. Furthermore, it records the sleep stage distribution, frequency of arousals during sleep, number of apneas or hypopneas, time taken to fall asleep, and sleep efficiency (total sleep time/total recording time). The AHI is also obtained by using PSG. The sleep stages verified by using PSG are divided into non-rapid eye movement (NREM) sleep and REM sleep. NREM sleep is subdivided into stages N1, N2, and N3, depending on the depth of sleep, with stage N3 being the deepest sleep, and REM sleep is indicated as stage R on a PSG image. Each sleep stage is determined on the basis of the EEG pattern results specific to each stage.

The substantial increase in N1 in the patient of this case report, which is likely to occur in OSA and other sleep disorders, indicated that he had a sleep disorder. The AHI score of 84.3 indicated a severe OSA level, and the oxygen saturation (minimum 70%) was considerably lower than normal.

PSG provides substantial sleep-related data, and it is useful for the assessment and treatment of sleep apnea. However, PSG is difficult to conduct because it involves an examiner’s constant monitoring of the subject, and the procedure is technically complex. The high cost of the examination is also a limitation. Additionally, the subject and examiner should follow the schedule of admission at around 8 p.m. and discharge at approximately 7 a.m. the next morning because the sleep study is performed during the entire period of sleep.

In response to these limitations of PSG, the PMD, which provides less information but is more cost-effective and less complicated, has been developed and used for sleep research. The AASM guidelines on the use of PMDs [42], since the time of their publication, have been used to examine subjects suspected of moderate or severe OSA; the U.S. Centers for Medicare and Medicaid Services (CMS) recognize insurance coverage for portable monitoring-based sleep disorder diagnoses [43]. PMDs measure the subject’s respiratory status, heart rate, oxygen saturation, and sleeping positions. Although PMDs are also used throughout the sleeping hours, unlike PSG, an overnight stay in a sleep laboratory or scheduling for admission is not necessary because the test can be performed at home. Further, an examiner is not needed to monitor the patient during sleep. Despite such financial and practical advantages, the PMD has the drawbacks of lower reliability because of the lack of a monitoring technician, unless the subjects use the test device correctly, and lower accuracy in measuring the time taken to fall asleep, thus resulting in a possible deviation from the AHI score measured by using PSG.

CPAP is the most effective therapy for OSA. During sleep, the patient wears a mask connected to a CPAP machine that continuously blows air into the patient’s upper airway and increases the intrapharyngeal positive pressure; therefore, the soft palate is prevented from sagging and the upper airway is maintained open because of the pressure. Some patients require a great deal of time and sufficient effort to become accustomed to this therapy, but it has proven to be effective in improving the objective and subjective sleep quality and the symptoms of cardiovascular disorders [11]. In mild cases of OSA, patients can be recommended conservative treatment prior to proceeding to CPAP, such as weight reduction and sleeping in a lateral decubitus position. Other therapies with proven efficacy are upper airway surgery or treatment by using an oral appliance (OA). The best therapy for moderate and severe OSA is CPAP [44,45]. Although upper airway surgery or OA therapy is recommended for patients who have difficulty adapting to the CPAP machine or for whom this therapy cannot be easily applied, several studies [46,47] have revealed that these methods are not as effective as CPAP.

Sleep disorders and excessive daytime sleepiness are associated with occupational and industrial accidents [48,49]. OSA is a risk factor for car accidents [50], and many studies have demonstrated a higher accident rate among sleep apnea patients compared to the general population [51,52]; patients with an AHI score ≥ 10 had a 6.3-fold higher accident rate compared to the general population [53]. Furthermore, Garbarino et al. reported that occupational drivers have a higher prevalence of OSA than the general population, and the correlation between commutation car accidents and daytime sleepiness in shift workers was prominent [54]. The patient described in this report caused a car accident while driving within the premises of his workplace, and, similar to his case, numerous occupations involve driving during work. OSA-associated car accidents can be extended to driving situations outside the working environment, for example, in traffic during commuting and business trips. A car accident can be fatal to the driver and the occupants of the vehicle as well as to those in other cars involved in the accident. Further, the damage can be far greater if a commercial vehicle driver transporting many passengers or a large quantity of freight causes an accident compared to cases of individual drivers.

According to Korean industrial accident statistics, while the number of industrial accidents per year (in thousands) decreased from 8.49‰ in 2004 to 6.50‰ in 2011, the frequency of accidents has stayed above 90,000 since 2007, which was higher than the 87,033 cases recorded in 2004. Although the sectors of transportation, warehousing, and communication services, to which occupational drivers belong, showed a slightly decreasing trend in the number of industrial accidents per year in thousands, from 7.79‰ in 2004 to 5.87‰ 2011, these values do not include car accidents that occurred in other occupational groups. This suggests that the number of driving-related accidents in industrial settings is actually higher. Given that sleep disorders including OSA may be responsible for such accidents, setting regulations regarding workers’ sleep disorders will greatly contribute to preventing potential occupational accidents associated with sleep disorders.

Many countries have established various institutional regulations regarding OSA. The Federal Aviation Administration (FAA) requires an aviation medical examiner to confirm the efficacy of therapy and treatment compliance for pilots with OSA. The pilot’s return to work is postponed if these factors are found to be inadequate. South Korea also has institutional regulations regarding sleep disorders in aviation crewmembers. If a pilot is diagnosed with a mild or severe case of OSA (≥ 15) by using PSG, he/she should undergo CPAP or surgical treatment, as judged appropriate, and an aviation physician can issue an assessment of conditional fitness for work only if the ESS is improved to < 8 points. Additionally, if mild (5 < AHI score < 15) OSA is diagnosed by using PSG, an aviation physician can issue a fitness for work certificate when the ESS is < 8.

The National Highway Traffic Safety Administration specifies the driver fitness medical guideline criteria as follows: A driver is deemed unfit for driving if he/she has been diagnosed with OSA with symptoms of daytime sleepiness or an AHI score ≥ 20 and cannot overcome the symptoms of daytime sleepiness with therapy or lower his/her AHI score to ≤ 19 [55]. Most American states have federal regulations regarding a driver’s medical fitness, especially California, Texas, and Maryland, where a driving limit is imposed if sleep apnea cannot be controlled. Moreover, the U.S. Congress has passed a bill so that the new criteria for apnea diagnosis to be set by the Federal Motor Carrier Safety Administration (FMCSA) will be established as administrative law, therefore, legalizing its regulation.

In Europe, more than 10 countries, including the United Kingdom, have determined OSA to be a disease subject to restrictions for a driver’s license. The Driver & Vehicle Licensing Agency of the United Kingdom imposes driving restrictions on those who have excessive daytime sleepiness symptoms such as OSA, and drivers of private vehicles are prohibited from driving until their symptoms are resolved. Occupational drivers can apply for a reissuance of their driver’s license once the symptoms are controlled and if they have shown compliance over a long period of time, and annual license reassessment is mandatory [56]. However, South Korea has no concrete assessment criteria or regulations regarding sleep disorders of commercial and private vehicle drivers, and the establishment of assessment criteria for sleep disorders and other diseases is needed.

In 2006, recommendations regarding the driving fitness for work assessment for commercial vehicle drivers suspected of OSA were issued according to the results of the joint research conducted by the American College of Chest Physicians, the American Association of Occupational and Environmental Medicine, and the National Sleep Foundation [57]. The recommendations have the following specifications: commercial vehicle drivers diagnosed with OSA that is confirmed by using PSG should undergo CPAP therapy as soon as possible; the therapy shall be considered effective when the AHI score is reduced to ≤ 5; in patients with a 5 ≤ AHI score ≤ 10, the therapy shall be considered effective by comparing the baseline values and treatment outcomes; after 2–4 weeks of treatment, a specialized physician shall assess the treatment efficacy and compliance of the driver, and the return to work shall depend on the assessment results; the driver shall be subjected to a reassessment of treatment compliance within 3 months of return to work, and, thereafter, the driver must comply with an annual follow-up visit to check the subjective symptoms and CPAP therapy maintenance.

Although the patient in the current case report was not a commercial vehicle driver, the accident occurred during driving and the above-mentioned recommendations for the assessment of fitness for work were applicable to his condition. He was diagnosed with OSA by using PSG, and required CPAP therapy and follow-up to monitor the AHI score reduction and improvement in his subjective symptoms.

In Korea, night workers will be considered for special medical examinations as a separate group starting from 2014 onward. Examination items related to sleep disorders such as the insomnia index, daytime sleepiness, and sleep quality have been established, and the Insomnia Severity Index (ISI), ESS, and Pittsburgh Sleep Quality Index (PSQI), will be implemented as assessment tools for these 3 items, respectively. A warning will be issued if all of these sleep assessment tools determine that the case is abnormal (ISI score ≥ 15, PSQI score ≥ 6, and ESS score ≥ 10) or sleep disorder symptoms requiring proper interventions are observed in the second in-depth interview. Cases in which the results of all 3 assessment tools are serious (ISI score ≥ 22, PSQI score ≥ 6, and ESS score ≥ 15) will be considered to have a sleep disorder. Implementing such obligatory health examinations will deliver basic data for research studies on sleep disorders in Korean workers, a topic that has not attracted much attention so far. However, the fact that the assessment tools to be applied in special medical examinations are self-administered questionnaire surveys is their common limitation in the screening for sleep disorders. Furthermore, no clear regulations or recommendations exist regarding the application of PSG, despite its critical role in the diagnosis, therapy, and assessment of fitness for work of employees suspected of having sleep disorders.

Given that untreated OSA among the working-age adult population is a disease that can become an enormous public health burden, greater interest and support of health authorities are necessary for the diagnosis and treatment of this disorder. Occupational and environmental medicine physicians will have to play a vital role in conducting special medical examinations of high-risk workers with OSA and in the preparation of assessment guidelines.

## Competing interests

The authors declare that they have no competing interests.

## Authors’ contributions

DHK and SH interviewed and wrote the article. JYR and BCS searched and assisted the related references. SL, CKL and YWK supported and advised medical view. All of the authors read and approved the final manuscript.
